# Supplementary material for: Causal interplay between lactose intolerance and gut microbiota: a combined bidirectional Mendelian randomization and in vivo validation study
Source: Front Nutr. 2026 Jun 1;13:1803337. doi: 10.3389/fnut.2026.1803337 (PMC13265576; doi:10.3389/fnut.2026.1803337)
Supplement: Supplementary file 1 [file Data_Sheet_1.zip › supplementary materials/Forward/leave one out cross validation/ebi-a-GCST90027550.finngen_R12_E4_LACTONAS.pdf]

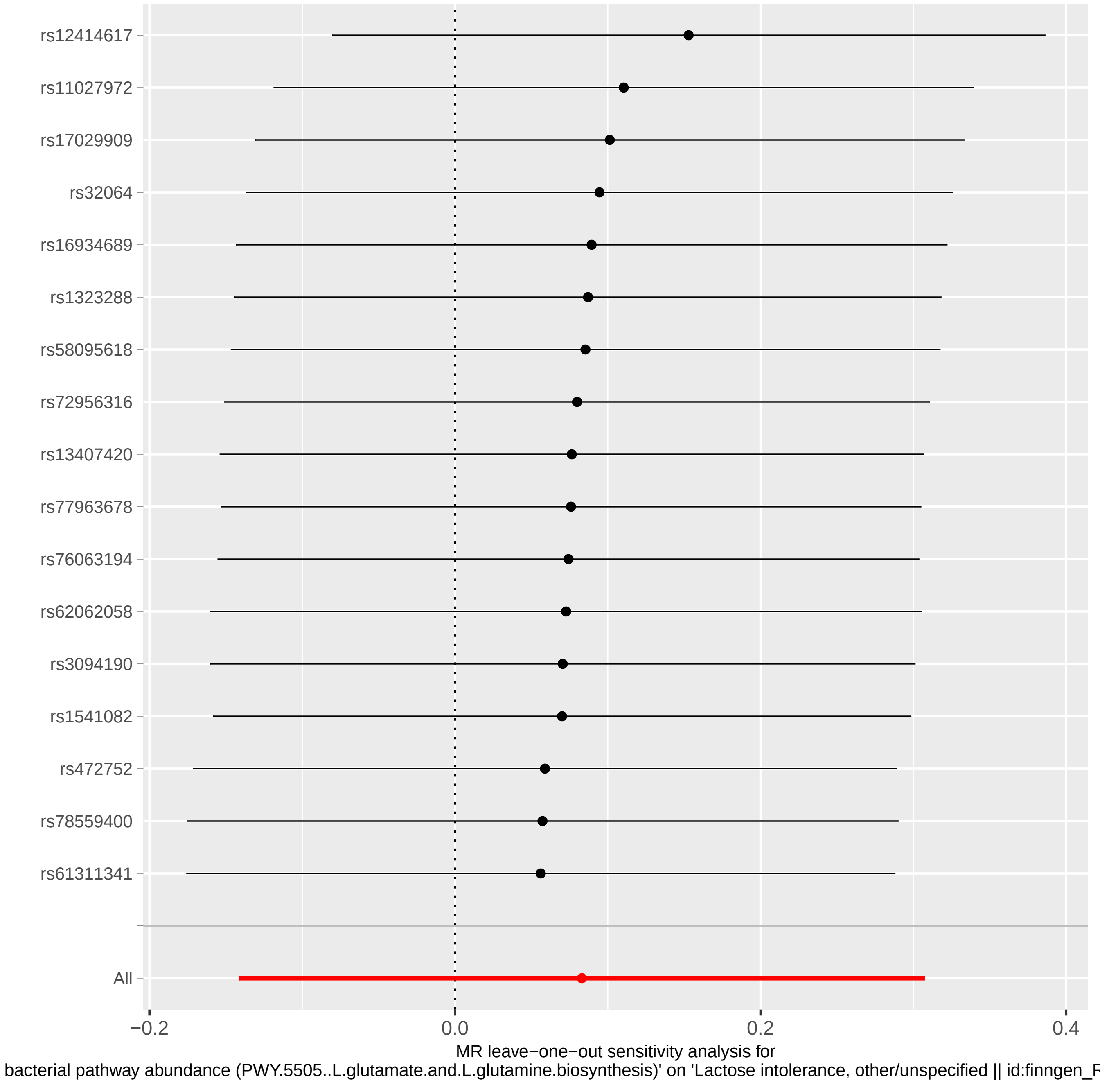
rs12414617
rs11027972
rs17029909
rs32064
rs16934689
rs1323288
rs58095618
rs72956316
rs13407420
rs77963678
rs76063194
rs62062058
rs3094190
rs1541082
rs472752
rs78559400
rs61311341
All
−0.2
0.0
0.2
0.4
MR leave−one−out sensitivity analysis for
bacterial pathway abundance (PWY.5505..L.glutamate.and.L.glutamine.biosynthesis)' on 'Lactose intolerance, other/unspecified || id:finngen_R
